# Objective and subjective evaluation of tear film in machine carpet weavers

**DOI:** 10.1002/1348-9585.12237

**Published:** 2021-06-03

**Authors:** Fatemeh Estarki, Amir Asharlous, Ali Mirzajani, Jamileh Abolghasemi

**Affiliations:** ^1^ Department of Optometry School of Rehabilitation Sciences Iran University of Medical Sciences Tehran Iran; ^2^ Department of Biostatistics School of Public Health Iran University of Medical Sciences Tehran Iran

**Keywords:** blink rate, carpet weaver, dry eye, ocular staining, tear deformation time, tear film

## Abstract

**Objectives:**

The present study was conducted to evaluate the status of tear film objectively and subjectively in machine carpet weavers.

**Methods:**

In this cross‐sectional study, machine‐made carpet weavers were compared with the controls who were selected from people working in other parts of the factory except for the production. A complete evaluation of ocular health was done for all participants. The blink rate, tear deformation Time (TDT), and ocular staining were evaluated as an objective assessment and the ocular surface disease index (OSDI) was used for a subjective assessment of the tear film status. The results were compared between the two groups using the SPSS software.

**Results:**

The results of 46 weavers (mean age: 38.43 ± 6.10 years) and 46 controls (mean age: 33.20 ± 8.40 years) were analyzed. The mean of blink rate and OSDI score were significantly higher in weavers (Blink rate: 20.67 ± 4.18 blink/min, OSDI: 22.59 ± 9.51) in comparison with controls (Blink rate: 14.00 ± 3.30 blink/min, OSDI: 6.22 ± 4.78, *P* < .001). The mean TDT value of the weavers was significantly lower compared with the controls (10.27 ± 3.01 and 16.58 ± 4.18 s respectively, *P* < .001). Ocular surface staining was seen among 60.9% of weavers while there was 6.5% in the controls (*P* < .001). Based on the TDT test and OSDI results together, the percentage of dry eye in the weavers was 43.5% and that in the non‐weavers was 2.2%, which showed that the relationship between weaving and dry eyes was statistically significant (*P* < .001).

**Conclusions:**

The results indicate that increased symptoms and decreased tear stability in weavers compared with non‐weavers lead to more tear film abnormalities in these individuals.

## 1 INTRODUCTION

The carpet weaving is an important part of the economy of Iranian society.^1^ In recent decades, although handmade carpet still has high popularity in global markets, in the domestic market, due to its cheaper price, flexibility in size, design, and color, machine‐made carpet has replaced the handmade one.^2^ Carpet weaving operation is considered as a precise task, for the knots are very fine and close together, and color identification is very important.^3^ If one knot loses its density, the supervisors must discover the defect during the inspection.^4^ High visual accuracy and constant close attention cause visual fatigue for weavers.^5^ Eye fatigue is one of the common visual complaints associated with dry eye.^6^

Dry eye disease (DED) is caused by many internal and external factors that can negatively affect the composition and stability of tear film.^7^ The tear film as the outermost layer of the ocular surface is the interface between the eye and the environment.^8^ Precorneal tear film stability is critical for ocular health, mainly because it nourishes, lubricates, and protects the ocular surface.^8^, ^9^

Occupational and environmental risk factors alter the stability of the precorneal tear film and can lead to the drying of the ocular surface.^10^ On the other hand, dry eye symptoms affect ocular comfort, health, and quality of life^11^ and have a negative impact on activities of daily living and work productivity.^12^

Some previous studies determined the relationship between refractive errors and ocular component values in hand‐made carpet weavers^5^ and compared refractive errors between a group of carpet weavers and a non‐weavers group.^13^ These studies concluded that carpet weaving had a strong correlation with myopia and ATR astigmatism. Also, refraction in carpet weavers had a significant relation to the ocular components.

Dry eye disease can decrease work productivity and have a negative impact on daily roles.^12^, ^14^ So far, no study has been done on the tear film of the weavers. This study focuses on the tear film, which plays a vital role in maintaining healthy visual function. This present study is conducted to evaluate tear film changes objectively by using tear deformity time test, blink rate, ocular staining, and subjectively by using a questionnaire in Kashan machine carpet weavers and finally to compare data between carpet weavers and non‐weavers group. The information obtained from the tear film evaluation was also used to assess the prevalence of dry eye in these individuals.

## 2 METHODS

### 2.1 Study design and data collection

This cross‐sectional study was conducted at Matini hospital, Kashan, the northern part of Isfahan province, Iran. Four machine‐made carpet weaving factories were selected from four different parts of the city based on the easy approach and willingness of employees. Two groups of the weavers and non‐weavers were chosen as study groups. The weavers group was chosen from weavers who worked at the production department in Kashan machine carpet factories. The control group was selected from people who were near to the production department but did not have knitting work, long‐term attention on close work, and close contact with the weaving machine. It was also noted that people who are selected as controls do not work with computer displays on a daily basis.

The inclusion criteria for subjects were 1 year of work experience or more, the age of 20‐45 years, absence of anterior ocular pathology based on slit‐lamp examination, no history of ocular surgery, no systemic disease that causes dry eye, no use of systemic drugs such as antihistamines and anticholinergics, which cause dry eye, no use of medicine for dry eyes until now, and no use of contact lenses. Employees who were unable to perform tests were excluded from the study.

### 2.2 Ethical approval

Before the examination, written informed consent was obtained from the participants. All the principles of the Helsinki declaration were respected in the various stages of this research. The study was approved by the Ethical Committee of Iran University of Medical Sciences (approval ID IR.IUMS.REC.1398.1357).

### 2.3 Examinations

First, initial examinations were performed to consider the inclusion criteria. Measurements were taken for all variables in the same place. The demographic characteristics and work experience of the participants were recorded. When taking the history, the number of blinks per minute was measured by direct observation using a timer. Subjects were unaware of the blink measurements. The ocular surface disease index (OSDI) questionnaire was completed by each subject to evaluate the subjective ocular symptoms.^15^, ^16^ The tear deformation time test (TDT) was performed using the Javal–Schiotz keratometer (Haag‐Streit AG) for non‐invasive tear stability evaluation. The vertical mode of the keratometer with a 2‐diopter interval between the red and green catoptric images was used. The time between the last blink and the occurrence of the mire image distortion was measured by a stopwatch. This procedure was repeated for each eye three times in succession, and the mean value of them was recorded as the TDT result.^17^ To evaluate ocular staining, a single drop of unit dose saline is instilled onto a fluorescein strip. The lower lid of each eye is then pulled down and the strip is tapped onto the lower tarsal conjunctiva. The staining patterns are observed using a slit‐lamp.

### 2.4 Definitions and diagnostic criteria

To analyze the OSDI, the score of each subject was calculated based on the following formula: OSDI = [(sum of scores for all questions answered) × 100]/[(total number of questions answered) × 4]. For classification of the intensity of dry eye based on the OSDI, a value of 0‐12, 13‐22, 23‐32, and more than 33 was considered as normal, mild dry eye, moderate dry eye, and severe dry eye, respectively.^15^, ^16^

According to the Tear Film and Ocular Surface Society (TFOS) Dry Eye Workshop (DEWS) II reports for dry eye diagnosis, we used TDT for non‐invasive assessment of TBUT and OSDI questionnaire for subjective evaluation of the dry eye.^16^ A result of OSDI scores ≥ 13 and TDT < 10 s together was considered as dry eye. For the scoring of the ocular staining, the Oxford schema was utilized.^18^

### 2.5 Statistical analysis

Statistical analysis was performed with SPSS Version 22 statistic software package. The normality of each of the parameters' distribution was checked using the Kolmogorov–Smirnov normality test. Mean and standard deviation for the variables were reported. The blink rate, TDT values and OSDI scores between groups were compared using an independent *t*‐test. The *χ* ^2^ test was used to analyze differences among the groups concerning fluorescein staining and dry eye disease. Analysis of covariance (ANCOVA) was used to adjust for the selected covariate (age and working hours) to control for potentially confounding factors. The Pearson correlation was used to investigate relationships. A *P*‐value of <.05 is statically significant.

## 3 RESULT

The study was done on 46 carpet weavers and 46 non‐weavers. The mean age of the weavers and non‐weavers was 38.43 ± 6.109 (rang: 25‐45) and 33.20 ± 8.403 (rang: 20‐45) years, respectively (*P* = .001). All the study participants were males because all machine‐made carpet weavers in Kashan are male. As the information about the two eyes was correlated, the data of one eye (the eye with poorer tear test values) were selected for the analysis (*P* < .001).

Table 1 presents the mean TDT, OSDI scores, and blink rate. The mean TDT value of the weavers was significantly lower compared with the controls (10.27 ± 3.01 and 16.58 ± 4.18 s respectively, *P* < .001). The mean blink rate was significantly higher in weavers (20.67 ± 4.18 blink/min) in comparison with controls (14.00 ± 3.30 blink/min, *P* < .001).

**TABLE 1:** Mean and SD of TDT, OSDI, and blink rate in both groups

Variables	Case (n = 46), Mean ± SD	Control (n = 46), Mean ± SD	*P*‐value
TDT (s)	10.27 ± 3.01	16.58 ± 4.18	<.001
OSDI	22.59 ± 9.51	6.22 ± 4.78	<.001
Blink rate (blink/min)	20.67 ± 4.18	14.00 ± 3.307	<.001

Abbreviations: OSDI, ocular surface disease index, SD, standard deviation; TDT, tear deformation time.

**FIGURE 1:**
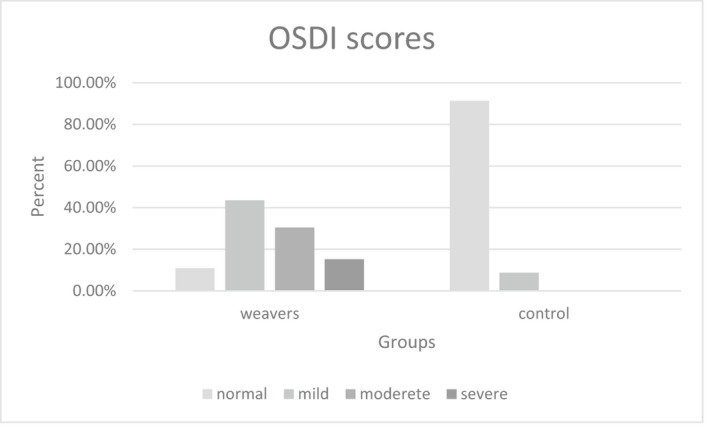
The severity of ocular surface disease index disorder in both groups

The mean OSDI was 22.59 ± 9.51 in weavers and 6.22 ± 4.78 in the control group (*P* < .001). The severity of dry eye in both groups based on OSDI results is presented in Figure 1. Severe OSDI disorder was significantly higher in weavers.

According to Table 2, staining of the ocular surface was seen among 60.9% of weavers while there was 6.5% in the control group. Weavers showed more ocular staining. When a Chi‐square test was done, the difference between groups was found to be statistically significant (*χ* ^2^ = 23.192; *P* < .001).

**TABLE 2:** The percentages of different ocular staining grades obtained from both studied groups

	Weavers (n = 46)	Non‐weavers (n = 46)
Grade 0	39.10%	93.50%
Grade 1	47.80%	6.50%
Grade 2	13.00%	0%

The effect of age, working time and working experience (with a criterion of at least 1 year of work experience) as confounding factors on each of the variables was evaluated. These factors had no significant effect on the variables. Results after adjustment for confounding factors showed that all indices were worse in weavers (*P* < .001).

To investigate dry eye in both groups, the data obtained from the OSDI questionnaire and TDT were assessed. The results of the study showed that 43.5% of weavers and 2.2% of the non‐weavers had dry eyes. This difference was significant (*P* < .001).

## 4 DISCUSSION

To the best of our knowledge, this is the first study to attempt tear film evaluation in machine‐made carpet weavers. This study focused on the comparison of tear film between weavers and non‐weavers objectively and subjectively. As mentioned earlier, the mean OSDI score for the weavers group was about four times higher than that in the control group, which is a statistically and clinically significant difference. Based on the studies performed, the presence of ocular symptoms and the ability to accurately quantify them with a validated questionnaire is an important tool for dry eye detection and monitoring its progression.^16^, ^19^ Considering the results of the OSDI questionnaire, it could be stated that the severity of dry eye in weavers was higher than that in the control group (Figure 1).

Tear film stability assessment ideally requires a non‐invasive methodology that evaluates the temporal changes of the tear in an interblink interval in an objective manner.^16^ For this purpose, we used the TDT technique as a non‐invasive assessment of tear film break‐up time.^17^ The TDT values were also worse in weavers which indicated that they have a less stable tear film. The difference in TDT results between the two groups was statistically significant. The results of the TDT strongly supported the results of subjective assessments of dry eye, indicating dry eye in a high percentage of weavers.

In this study, the rate of blinking per minute was found to be significantly higher in the weavers as compared with the control group. A negative correlation was observed between blink rate and TDT values, such that a higher blink rate was associated with decreased tear deformation time. An increase in the blink rate may be a compensatory mechanism for reduced tear stability.^20^ Patients with DES can usually clear a distorted image temporarily by increasing blinking to redistribute the tear film over the ocular surface.^21^ Therefore, blink count testing may be considered a reliable indicator of dry eye risk.^22^

According to the Tear Film and Ocular Surface Society (TFOS) Dry Eye Workshop (DEWS) global dry eye definition, inflammation is a key pathogenic factor in DED.^23^ Ocular surface damage due to inflammation can be demonstrated with the fluorescein staining method.^8^ The TFOS has considered staining as an important aspect in the clinical analysis of DED.^16^, ^24^ The results of the staining in conjunction with other tests performed in this study indicate that the corneal and conjunctival superficial epithelial cells of the weaver may be damaged.

Regarding all tear analyses, it can be stated the prevalence of dry eye was higher in weavers compared with non‐weavers. As no study has evaluated tear film in weavers, a comparison of the results with similar studies is not possible. The important question is, “Why do weavers have abnormal tear film?” One possible hypothesis may be high visual demanding and concentration during the weaving process. In this procedure, weavers carefully check the color and quality of the carpet and match the carpet weaving with the patterned design on the computer display of the machine. Several studies have reported that performing tasks that require too much attention and intensive use of the eyes such as computer and cognitive work are responsible for several eye‐related symptoms such as pain and tiredness in the workplace.^9^, ^25^, ^26^ It is known that demanding task content decreases blinking and induces tear film alterations that result in tear film instability.^27^, ^28^ An elevated blink rate in weavers when they are not working and reduced visual demand maybe a compensatory mechanism for reduced tear stability.^20^, ^29^

Another possible reason for tear film abnormalities in weavers may be the effect of environmental factors. Several external environmental factors have been suggested to impact tear film, such as temperature,^9^ air conditioning, low humidity,^30^ air pollution,^31^ and other atmospheric irritants.^32^ No studies have been conducted on the quality of the working environment of industrial factories producing machine‐made carpets, but previous studies have shown poor ventilation and lack of fresh air in hand‐woven carpet workshops.^33^ The composition of the particles from the wool and synthetic fibers released into the atmosphere irritates the ocular surface and causes symptoms in textile workers.^4^, ^33^, ^34^ Also, lighting is not sufficient in many hand‐made weaving workshops, which results in considerable eye strain.^3^, ^35^ Therefore, the second hypothesis to explain tear film abnormality in weavers are environmental triggers.

## 5 CONCLUSION

Generally, based on the results of the present study, it can be concluded that the abnormalities of the tear film are higher in weavers compared with non‐weavers. The result in the weavers group showed significantly increased symptoms, blink rate, and ocular staining and decreased TDT values. The most important findings in the study were the difference in the OSDI score and staining between the two groups, indicating more frequent dry eye complaints and damage to the ocular surface of machine‐made carpet weavers. The work environment of the weavers should be improved to decrease dry eye symptoms and to enhance ocular surface health. We suggest further studies on the environmental health status and nature of dry eye in machine‐made carpet factories.

## DISCLOSURE

*Approval of the research protocol*: All the principles of the Helsinki declaration were respected in the various stages of this research. The study was approved by the Ethical Committee of Iran University of Medical Sciences (approval ID IR.IUMS.REC.1398.1357). *Informed consent*: All participants of this study gave informed consent. *Registry and the registration no. of the study*: N/A. *Animal studies*: N/A. *Conflict of interest*: None declared.

## AUTHOR CONTRIBUTIONS

Fatemeh Estarki conceived the ideas and collected the data. Dr Amir Asharlous was a scientific advisor in all stages of the study. Dr Ali Mirzajani supervised all stages of the study and led the writing. Dr Jamileh Abolghasemi analyzed the data statistically.

## References

[1] Niazi M , Chitsazian A‐H . Role of Job Culture and Working Home‐Outside in Carpet Industry. Vol 3. Journal Scientific Goljaam; 2007. http://goljaam.icsa.ir/article‐1‐370‐en.html. Accessed January 12, 2021.

[2] Rahimi S , Fallahnezhad MS , Saleh Owlia M , Hossein AM . Investigation of Customer Priorities for Machine Made Carpet Through Conjoint and Cluster Analysis (Case Study in Yazd, Iran). Vol 6. University of Sistan and Balouchestan. 2014.

[3] Choobineh A , Shahnavaz H , Lahmi M . Major health risk factors in iranian hand‐woven carpet industry. Int J Occup Saf Ergon. 2004;10(1):65‐78. 15028195 10.1080/10803548.2004.11076596

[4] Ghvamshahidi Z . The linkage between Iranian patriarchy and the informal economy in maintaining women’s subordinate roles in home‐based carpet production. Womens Stud Int Forum. 1995;18(2):135‐151.

[5] Yekta A , Fotouhi A , Hashemi H , et al. Relationship between Refractive Errors and Ocular Biometry Components in Carpet Weavers. Vol 22. Journal of Current Ophthalmology; 2010. www.SID.ir. Accessed January 12, 2021.

[6] Koh S . Mechanisms of visual disturbance in dry eye. Cornea. 2016;35(11):S83‐S88. 27583799 10.1097/ICO.0000000000000998

[7] Yazdani M , Elgstøen KBP , Rootwelt H , Shahdadfar A , Utheim ØA , Utheim TP . Tear metabolomics in dry eye disease: a review. Int J Mol Sci. 2019;20(15):3755. 10.3390/ijms20153755 PMC6695908 31374809

[8] De Pinho Tavares F , Fernandes RS , Bernardes TF , Bonfioli AA , Carneiro Soares EJ . Dry eye disease. Semin Ophthalmol. 2010;25(3):84‐93. 20590418 10.3109/08820538.2010.488568

[9] Willcox MDP , Argüeso P , Georgiev GA , et al. TFOS DEWS II tear film report. Ocul Surf. 2017;15(3):366‐403. 28736338 10.1016/j.jtos.2017.03.006 PMC6035753

[10] Wolkoff P . Dry eye symptoms in offices and deteriorated work performance – a perspective. Build Environ. 2020;172:106704.

[11] Tounaka K , Yuki K , Kouyama K , et al. Dry eye disease is associated with deterioration of mental health in Male Japanese University Staff. Tohoku J Exp Med. 2014;233(3):215‐220. 25055758 10.1620/tjem.233.215

[12] McDonald M , Patel DA , Keith MS , Snedecor SJ . Economic and humanistic burden of dry eye disease in Europe, North America, and Asia: a systematic literature review. Ocul Surf. 2016;14(2):144‐167. 26733111 10.1016/j.jtos.2015.11.002

[13] Yekta A , Hashemi H , Ostadimoghaddam H , et al. Impact of carpet weaving on refractive errors. Iran J Ophthalmol. 2011;23(4):29‐36. http://irjo.org/article‐1‐544‐en.html. Accessed July 18, 2019.

[14] Patel VD , Watanabe JH , Strauss JA , Dubey AT . Work productivity loss in patients with dry eye disease: An online survey. Curr Med Res Opin. 2011;27(5):1041‐1048. 21417803 10.1185/03007995.2011.566264

[15] Schiffman RM , Christianson MD , Jacobsen G , Hirsch JD , Reis BL . Reliability and validity of the ocular surface disease index. Arch Ophthalmol. 2000;118(5):615‐621. 10815152 10.1001/archopht.118.5.615

[16] Wolffsohn JS , Arita R , Chalmers R , et al. TFOS DEWS II diagnostic methodology report. Ocul Surf. 2017;15(3):539‐574. 28736342 10.1016/j.jtos.2017.05.001

[17] Asharlous A , Jafarzadehpur E , Mirzajani A , Khabazkhoob M . Comparing tear film stability prolongation evaluated by Javal‐Schiotz Keratometer and Slitlamp. Eye Contact Lens Sci Clin Pract. 2015;41(2):101‐106. 10.1097/ICL.0000000000000073 25503909

[18] Bron A , Evans V , Cornea JS . 2003 undefined. Grading of corneal and conjunctival staining in the context of other dry eye tests. *journals.lww.com*. https://journals.lww.com/corneajrnl/Fulltext/2003/10000/The_Height_and_Radius_of_the_Tear_Meniscus_and.8.aspx. Accessed December 14, 2019. 10.1097/00003226-200310000-00008 14508260

[19] Smith JA , Albenz J , Begley C , et al. The epidemiology of dry eye disease: report of the epidemiology subcommittee of the international Dry Eye WorkShop (2007). Ocul Surf. 2007;5(2):93‐107. 17508117 10.1016/s1542-0124(12)70082-4

[20] Nosch DS , Pult H , Albon J , Purslow C , Murphy PJ . Relationship between corneal sensation, blinking, and tear film quality. Optom Vis Sci. 2016;93(5):471‐481. 27104591 10.1097/OPX.0000000000000827

[21] Miljanović B , Dana R , Sullivan DA , Schaumberg DA . Impact of dry eye syndrome on vision‐related quality of life. Am J Ophthalmol. 2007;143(3):409‐415.e2. 17317388 10.1016/j.ajo.2006.11.060 PMC1847608

[22] Wolffsohn JS , Craig JP , Vidal‐Rohr M , Huarte ST , Ah Kit L , Wang M . Blink test enhances ability to screen for dry eye disease. Contact Lens Anterior Eye. 2018;41(5):421‐425. 29958779 10.1016/j.clae.2018.06.003

[23] Craig JP , Nichols KK , Akpek EK , et al. TFOS DEWS II definition and classification report. Ocul Surf. 2017;15(3):276‐283. 28736335 10.1016/j.jtos.2017.05.008

[24] Sullivan BD , Crews LA , Sönmez B , et al. Clinical utility of objective tests for dry eye disease. Cornea. 2012;31(9):1000‐1008. 22475641 10.1097/ICO.0b013e318242fd60

[25] Larese Filon F , Drusian A , Ronchese F , Negro C . Video display operator complaints: a 10‐year follow‐up of visual fatigue and refractive disorders. Int J Environ Res Public Health. 2019;16(14):2501. 10.3390/ijerph16142501 PMC6678724 31337021

[26] Thorud H‐MS , Helland M , Aarås A , Kvikstad TM , Lindberg LG , Horgen G . Eye‐related pain induced by visually demanding computer work. Optom Vis Sci. 2012;89(4):E452‐E464. 22366711 10.1097/OPX.0b013e31824c1801

[27] Portello JK , Rosenfield M , Chu CA . Blink rate, incomplete blinks and computer vision syndrome. Optom Vis Sci. 2013;90(5):482‐487. 23538437 10.1097/OPX.0b013e31828f09a7

[28] Wolkoff P , Nøjgaard JK , Troiano P , Piccoli B . Eye complaints in the office environment: precorneal tear film integrity influenced by eye blinking efficiency. Occup Environ Med. 2005;62(1):4‐12. 15613602 10.1136/oem.2004.016030 PMC1740860

[29] Himebaugh NL , Begley CG , Bradley A , Wilkinson JA . Blinking and tear break‐up during four visual tasks. Optom Vis Sci. 2009;86(2):E106‐E114. 19156014 10.1097/OPX.0b013e318194e962

[30] Cho HA , Cheon JJ , Lee JS , Kim SY , Chang SS . Prevalence of dry eye syndrome after a three‐year exposure to a clean room. Ann Occup Environ Med. 2014;26(1):1‐6. 25339991 10.1186/s40557-014-0026-z PMC4205474

[31] Stapleton F , Alves M , Bunya VY , et al. TFOS DEWS II epidemiology report. Ocul Surf. 2017;15(3):334‐365. 28736337 10.1016/j.jtos.2017.05.003

[32] Wolkoff P . “Healthy” eye in office‐like environments. Environ Int. 2008;34(8):1204‐1214. 18499257 10.1016/j.envint.2008.04.005

[33] Ozesmi M , Aslan H , Hillerdal G , Rylander R , Baris YI . Byssinosis in carpet weavers exposed to wool contaminated with endotoxin. Br J Ind Med. 1987;44(7):479‐483. 3620372 10.1136/oem.44.7.479 PMC1007864

[34] Fishwick D , Fletcher AM , Anthony C , Pickering C , McL Niven R , Faragher EB . Ocular and nasal irritation in operatives Lancashire cotton and synthetic fibre mills. Occup Environ Med. 1994;51(11):744‐748. 7849851 10.1136/oem.51.11.744 PMC1128098

[35] Ranjbarian M , Gheibi L , Hatami H , Khodakarim S . Lighting conditions and vision status in carpet weaving workshops and workers at the city of Takab in 2013. Iran J Ergon. 2015;2(4):11‐17. http://journal.iehfs.ir/article‐1‐146‐en.html. Accessed January 12, 2021.

